# Spinal Cord Stimulation Therapy for Phantom Limb Pain in the Left Lower Extremity 30 Years After Onset

**DOI:** 10.7759/cureus.92430

**Published:** 2025-09-16

**Authors:** Mika Sasaki, Hisashi Date, Hiroyuki Ito

**Affiliations:** 1 Anesthesiology, Oita University Hospital, Oita, JPN; 2 Anesthesiology, Sendai Pain Clinic Center, Sendai, JPN

**Keywords:** exercise therapy, mirror therapy, phantom pain, pulsed radiofrequency treatment, rehabilitation, spinal cord stimulation therapy (scs)

## Abstract

A male patient in his 70s underwent left hip disarticulation 34 years ago due to a malignant tumor in the left thigh. Phantom limb pain developed 30 years ago, with night pain worsening nine years ago. Despite increasing the codeine phosphate dosage and performing a caudal epidural block, his pain did not improve, leading him to visit our hospital. He experienced sharp, electric-like pain in the phantom left sole, with a Visual Analogue Scale score of 100 mm. The phantom limb sensation was in a flexed lower-limb position, and he was unable to move it voluntarily. Temporary pain relief was achieved through a lumbar epidural block. Rehabilitation was initiated, including mirror therapy and exercise therapy for areas outside the affected limb. Six months later, somatosensory sensation returned in the phantom limb, and pulsed radiofrequency was applied to the left L5 and S1 nerve roots, resulting in pain relief. However, pain recurred 10 months later. The patient continued exercise therapy, and after 12 months, voluntary movement of the phantom limb was achieved. Spinal cord stimulation (SCS) therapy was then performed. After the trial, phantom pain decreased, and insomnia caused by night pain improved, leading to implantation. In this case, effective treatment was achieved using SCS for phantom limb pain that had persisted for 30 years. Although there were concerns regarding the chronic nature of the phantom pain and the difficulty of achieving treatment success, rehabilitation combined with SCS appeared to enhance the treatment outcome.

## Introduction

Phantom limb pain is a condition in which patients experience pain in a body part that has been lost, either wholly or partially, after limb amputation [1]. It is reported to occur in approximately 64% of patients following amputation [2]. While no established nerve block treatment exists for phantom limb pain, some reports suggest the effectiveness of pulsed radiofrequency (PRF) [3,4]. Similarly, studies on spinal cord stimulation (SCS) for phantom limb pain have demonstrated positive results [5]. However, the number of patients included in these studies has been relatively small, and there remains insufficient evidence regarding which patients are most likely to benefit. Furthermore, mirror therapy, as part of rehabilitation, is known to alleviate pain, although the level of evidence is low [6].

In this case, we report the effectiveness of SCS therapy in a patient with phantom limb pain in the left lower limb that had persisted for 30 years.

## Case presentation

A male patient in his 70s, 176 cm in height and weighing 76.3 kg, underwent left hip disarticulation 34 years ago due to a malignant tumor in the left thigh. He subsequently used a prosthetic limb and remained pain-free. Phantom limb pain in the left lower limb appeared 30 years ago but was alleviated during work. Nine years ago, following retirement, he stopped using the prosthesis, and his pain gradually worsened. His local clinic prescribed codeine phosphate and performed caudal epidural blocks; however, persistent pain developed eight years ago. Despite increasing the codeine phosphate dosage, insomnia due to night pain persisted, leading to his referral to our hospital.

The left lower limb was amputated at the hip, and the patient did not use a prosthetic limb but ambulated with crutches. At the time of presentation, the pain was rated 100 mm on the Visual Analogue Scale (VAS), while persistent pain was reported as 3 out of 10 on the Numerical Rating Scale (NRS) for the phantom left sole. He described the pain as electric-like, lasting 30-60 minutes, and noted several nights per month of complete insomnia due to the pain. The length of the phantom limb was normal, with no shortening or retraction. The phantom limb was perceived in a flexed lower-limb position, with the sensation of being seated, but he was unable to move it voluntarily. No stump pain was present. The Hospital Anxiety and Depression Scale revealed an anxiety score (A) of 4/21 and a depression score (D) of 7/21, indicating no significant anxiety or depression.

**Figure 1:**
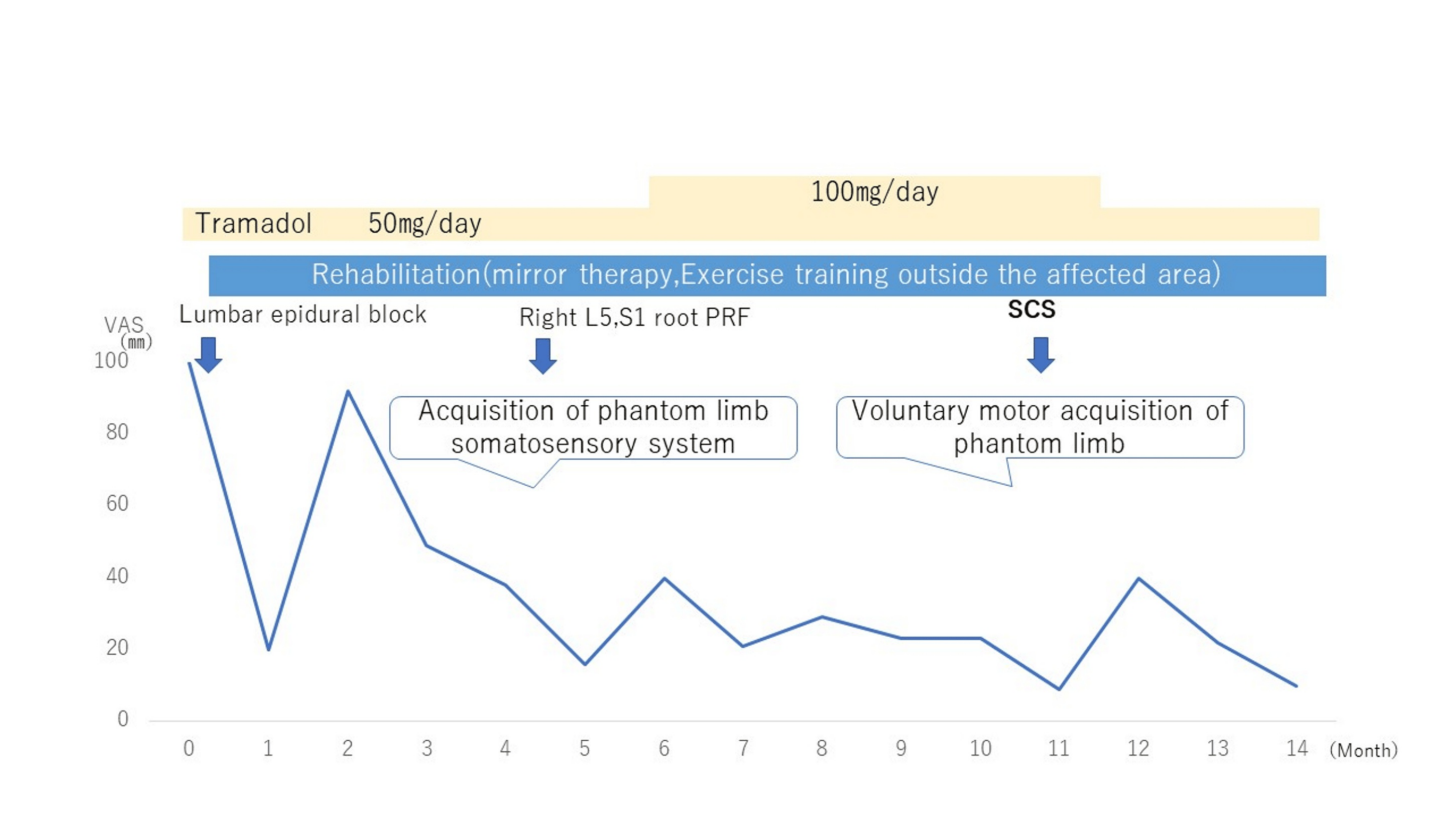
Progress after initial medical assessment PRF: pulsed radiofrequency; SCS: spinal cord stimulation

The patient’s medication was switched from codeine phosphate to tramadol 50 mg/day. At the initial visit, a lumbar epidural block was performed, resulting in temporary complete resolution of phantom limb pain. One week later, the VAS decreased to 17 mm, indicating significant pain relief (Figure 1). To achieve long-term pain relief, rehabilitation was initiated, including mirror therapy and exercise therapy for areas outside the affected limb. During mirror therapy, the image of the healthy limb was reflected in the mirror, and the patient repeatedly imagined the presence of the phantom limb. Additionally, decreased flexibility was observed in the non-affected limbs and trunk, and stretching, exercises, and gait training were performed once a week.

The line represents monthly changes in VAS scores (mm), while the arrows indicate the timing of nerve blocks and neuromodulation procedures. Rehabilitation was initiated at the first visit. PRF was applied to the L5 and S1 nerve roots when movement sensation of the phantom limb was obtained. SCS was introduced once voluntary movement of the phantom limb was achieved. Tramadol was administered from the first visit, and the dosage was reduced following the initiation of SCS.

**Figure 2:**
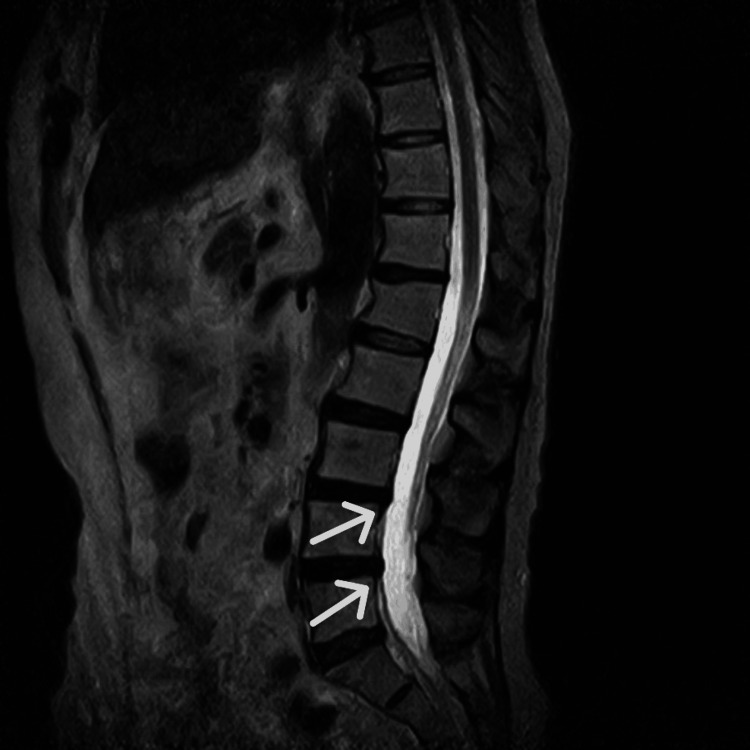
Sagittal T2-weighted MRI showing degenerative lumbar spondylosis and disc bulging at the L3/4 and L4/5 levels

**Figure 3:**
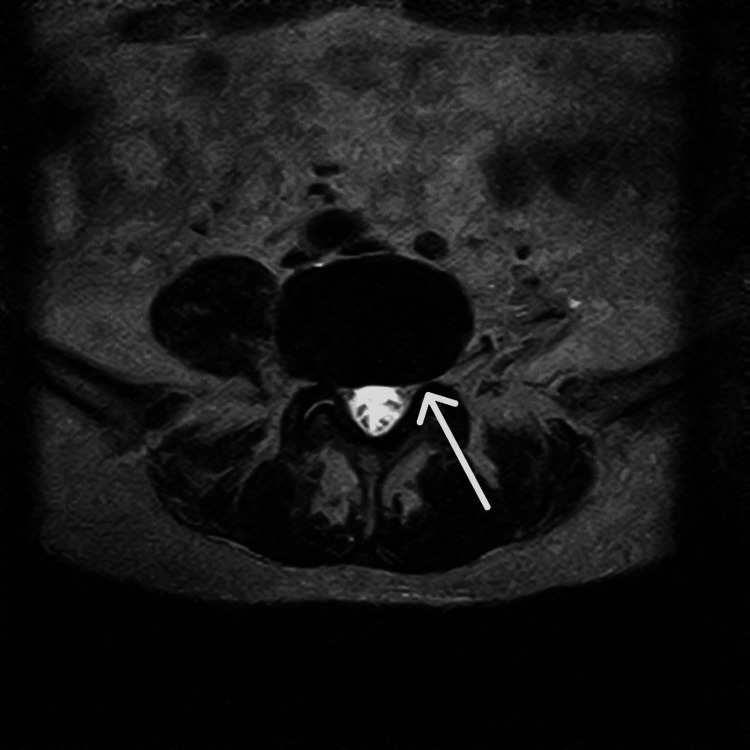
Axial MRI at the L4/5 level showing mild stenosis in the left L5 foraminal region

Lumbar MRI revealed degenerative lumbar spondylosis, disc bulging at the L3/4 and L4/5 levels, and mild stenosis in the left L5 foraminal region (Figure 2, Figure 3). Although voluntary movement of the affected limb was difficult, the patient had regained movement sensation in the phantom limb. PRF was therefore performed on the left L5 and S1 nerve roots. Stimulation of the left S1 nerve root evoked sensations throughout the phantom limb, particularly in the posterior lower leg, but did not produce stimulation in the most painful area of the phantom foot. During PRF of the left L5 nerve root, stimulation corresponded to the pain sites in the left sole, lateral lower leg, and first toe, suggesting that the origin of the phantom pain was at the L5 level. Pain relief was achieved after PRF, but recurrence occurred six months later.

Although the patient continued exercise therapy, after 10 months, he was able to achieve some voluntary movement of the phantom limb. Judging that the patient could now control the phantom limb, SCS was initiated through a surgical trial under local anesthesia. A bilateral epidural approach was performed at the L1/2 level, and two 16-contact SCS electrodes (Infinion™ CX 16, Boston Scientific, Marlborough, MA, USA) were implanted near the midline and left side, centered at the Th10/11 level. Electrode lead placement was confirmed by eliciting stimulation in the foot sole near the center of the lead.

The stimulation protocol included continuous use of FAST and Contour modes, with Tonic stimulation applied during pain episodes. After the trial, persistent pain decreased from NRS 2/10 to 1/10, and intense paroxysmal pain at night decreased from NRS 4/10 to 1/10, resulting in a significant reduction of phantom limb pain. Insomnia due to nighttime pain improved, and seven days after hospitalization, implantation of the spinal cord stimulator (implantable pulse generator) was performed under general anesthesia.

Following discharge, the patient initially experienced a temporary increase in VAS scores due to increased activity. However, the VAS decreased again with stretching, and the patient has since remained free from nighttime pain.

## Discussion

It has been reported that the incidence and intensity of phantom limb pain are related to how the pain is perceived, potentially due to a mismatch in the sensory-motor loop and plastic changes in the central nervous system. In this case, the patient stopped using the prosthesis, making it impossible to visualize the left lower limb, and the decreased motor sensation of the phantom limb likely contributed to the worsening pain. Therefore, the reduction in phantom limb pain may have been achieved through mirror therapy, which helped the patient regain lost somatosensation. Furthermore, in patients with a phantom limb, when no pain is present, performing mirror therapy to move the phantom limb activates the contralateral primary motor cortex and primary somatosensory cortex on functional MRI, similar to healthy individuals. However, in patients with phantom limb pain, activation in these areas is not observed [7]. This suggests that when the phantom limb’s form is close to normal and motor imagery is achieved, pain reduction is more likely.

There is no established nerve block treatment for phantom limb pain, although reports have shown the effectiveness of PRF applied to peripheral nerves and the dorsal root ganglion [3,4]. Similarly, studies on SCS for phantom limb pain have demonstrated positive results [5]. However, the number of patients included in these studies has been relatively small, and there remains insufficient evidence regarding which patients would benefit the most. In this case, the somatosensory input and motor imagery regained through mirror therapy may have enhanced the effectiveness of SCS, leading to a reduction in phantom limb pain.

Additionally, the patient’s decreased activity level due to retirement and the COVID-19 pandemic may have contributed to worsening phantom limb pain. It has been reported that movement in areas other than the pain site can reduce sensitivity to noxious stimuli, a phenomenon known as exercise-induced hypoalgesia (EIH) [8]. The exercise therapy performed on unaffected areas of the body, in this case, may therefore have contributed to pain reduction through EIH.

In this case, mild left L5 nerve root compression was observed on lumbar MRI, and pain relief was achieved through PRF of the left L5 nerve root. The evaluation of phantom limb pain based on physical findings is limited, and identifying the nerve root level or differentiating it from lumbar spinal lesions can be challenging. There have been reports of symptom improvement with spinal decompression in patients with nerve root involvement and phantom limb pain [9]. It is possible that the patient in this case had concomitant radiculopathy, and SCS may have been effective for both conditions.

## Conclusions

Phantom limb pain is often a treatment-resistant chronic pain condition that persists over long periods, posing a significant challenge in pain management. In this case, significant pain relief was achieved for approximately 30 years of refractory phantom limb pain through a multimodal approach combining physical therapy and SCS. The partial pain reduction observed during physical therapy prior to SCS implantation, along with the improvement following timely SCS application, suggests that the combination and timing of treatments are crucial for maximizing therapeutic effectiveness. These findings highlight the potential importance of integrating SCS with rehabilitation and suggest that multimodal strategies may be beneficial in managing chronic pain conditions such as phantom limb pain.
